# Trust as a Solution to Human Vulnerability: Ethical Considerations on Trust in Care Robots

**DOI:** 10.1111/nup.70020

**Published:** 2025-03-11

**Authors:** Mario Kropf

**Affiliations:** ^1^ Institute of Moral Theology, Faculty of Catholic Theology University of Graz Graz Austria

**Keywords:** artificial intelligence, care robots, ethics, trust, vulnerability

## Abstract

In the care sector, professionals face numerous challenges, such as a lack of resources, overloaded wards, physical and psychological strain, stressful constellations with patients and cooperation with medical professionals. Care robots are therefore increasingly being used to provide relief or to test new forms of interaction. However, this also raises the question of trust in these technical companions and the potential vulnerability to which these people then expose themselves. This article deals with an ethical analysis of the two concepts of trust and vulnerability in the context of care robotics. The first step is to examine what can be understood by vulnerability, focusing specifically on Misztal's three proposed types (relationships, future anticipation, past experiences). This strategy is often used as a starting point by authors and also seems relevant for the connection to the concept of trust. In a second step, these three types of human vulnerability are examined on the basis of a technical concept of trust. It is shown that (1) relationships and thus also interdependence can create additional options, (2) the anticipation problem with regard to the actions of others also makes responsibility transferable and (3) an explication of freedom is also associated with potential traumatic experiences. The final step brings together the previous considerations and makes it clear once again that trust in a care robot need not only be associated with vulnerability, but that vulnerability can also potentially be reduced, transferred and overcome.

## 1 Introduction

Trust is not only a philosophical concept associated with different ideas and consequences, but also seems to be attracting more attention than ever at the moment. This concerns both people working in science, for example, in the humanities, the IT sector or the neurosciences, as well as the general public or the people in a society (Campbell 2020, 110–111). In recent years, technologization, in particular, shaped by AI research, has made a significant contribution to the fact that the question of trust is increasingly being discussed. But what can be understood by this term and what is the connection between trust and vulnerability? This article deals with an ethical analysis of the concept of trust and the frequently described connection to vulnerability, using the example of AI‐based care robots. The first step is to discuss what can be understood by vulnerability. The types of relationships proposed by Misztal, the anticipation problem and the traumatic experience, form the starting point (Misztal 2012, 219–220). The second step analyzes the extent to which vulnerability can also be understood in a positive way, respectively the extent to which trust can contribute to a reduction, a transfer or an overcoming of one's own vulnerability. It is shown that trust in a technical helper can certainly counter human vulnerability, especially because the technical relationship of trust with a care robot opens up new options, responsibility is delegated and freedom can also be explicated. In a third step, the considerations made are summarized and it is pointed out that vulnerability is not to be understood exclusively negatively and that a plausible and ethically relevant connection to the concept of trust can be established.

## 2 Vulnerability

The term vulnerability is often associated with a particular susceptibility, an exceptional need for help or a disadvantaged position attributed to specific groups (Adger 2006, 268–269; Delor and Hubert 2000, 1557–1558; Pellegrino 1994, 483). It is therefore a combination of external (pressure, social exclusion, discrimination) and internal (inadequate processing, psychological and physical overload) factors that contribute to this situation or positioning (Nienaber et al. 2015, 569–570). The individuals in question find themselves in a precarious and vulnerable situation, which in most cases was not intentionally brought about. This non‐intentionality means that although these people often fall into a vulnerable group or a related categorization, they have not consciously worked towards being vulnerable—at least according to the intuitively plausible assumption. Beauchamp and Childress clarify this idea with reference to test subjects, for example, for clinical studies (Beauchamp and Childress 2019, 286–287). Due to their situation, these people could feel pressured to take part in experiments in order to improve their current situation of vulnerability. This could include economic disadvantages such as limited access to healthcare, a poor financial situation, lack of employment or malnutrition (Adger 2006, 270–271; Gaynor and Wilson 2020, 834–835; Plomp and Ballast 2010, 261–262). This first understanding of vulnerability thus also refers to socio‐cultural factors that describe individuals or groups as vulnerable, regardless of their humanity.

In agreement with Beauchamp and Childress, however, caution is required when making such an attribution (Beauchamp and Childress 2019, 287–288). Just because people belong to a certain group that is usually described as vulnerable does not necessarily mean that they feel the same way. Sometimes it is said that children, elderly people, prisoners or even pregnant women are automatically included under the label vulnerable (Blum et al. 2002, 28–29; Gaynor and Wilson 2020, 835–836; Kuran et al. 2020, 1–2; Mah et al. 2023, 7–8). However, this goes hand in hand with the ethically relevant aspect of self‐determination, or rather its restriction, because certain actions and decisions are attributed to these people as unreasonable and excessive demands (Callahan 1994, 485–486). In addition, stereotyping takes place on the basis of certain characteristics, conditions that have occurred or even specific stages of life, which in themselves by no means determine a person's vulnerability. Rather, the focus on the respective individual and their perception of themselves and others appears to be decisive. The definition of the *United Nations Office for Disaster Risk Reduction* (UNDRR) offers a good point of reference to realize that the term vulnerability is associated with many different aspects.

‘The conditions determined by physical, social, economic and environmental factors or processes which increase the susceptibility of an individual, a community, assets or systems to the impacts of hazards’.

UNDRR 2007

The causes described, which are decisive for the attribution of vulnerability and the associated risks, relate to socio‐economic factors. Kottow draws attention to the fact that this idea is associated with ‘[…] a specific and accidential condition to be diagnosed and treated […]’ (Kottow 2004, 284) and at the same time refers to considerations of justice theory. The disadvantages and, above all, unfavourable circumstances with which individuals or groups are confronted are usually due to social, cultural, disease‐related or religious factors (Gilpin 2022, 144–145; Schiavo 2022, 243–244). Apart from this, the subjective dimension of vulnerability, in the form of personal or group‐related consent, is addressed implicitly at most, and minimally not at all. At the same time, it becomes clear that targeted measures are needed in the health sector to reduce these potential vulnerabilities (Schiavo 2022, 243).

In a perspective contrary to the previous explanations, vulnerability is to be located in the nature of human beings themselves. This human *fragility* is therefore not only reflected in specific groups, but generally affects all individuals by virtue of their humanity (Kottow 2004, 282–283). Quepons emphasizes a relative character, according to which vulnerability depends on situational and person‐specific circumstances and is either an ‘[…] essential dimension in the constitution of embodiment […]’ (Quepons 2020, 1), or a disposition for trust. The former view is associated with a subjective perception of risks or one's own vulnerability in general, which integrates emotional aspects. The second form of vulnerability appears to be essential for trust and is considered in more detail below. Nienaber et al. differentiate between active and passive vulnerability, which certainly match Quepon's distinction. The first form (affective) is characterized by emotional aspects, interaction and appreciation of the respective counterpart. The second form (cognitive) corresponds to an assessment of certain behaviours and refers to the reliability of another individual (Nienaber et al. 2015, 575–576).

Misztal defines three types (Misztal 2012, 218–220) of vulnerability and also refers to Nussbaum. This classification is relevant for this article as it allows different dimensions of *human* vulnerability to be recognized. However, it should be kept in mind that the following types are an interpretation based on human interactions and therefore need to be reviewed with regard to their applicability to AI‐based care robots. The (1) dependence on others can be understood as our actions and decisions not taking place in a vacuum, but influencing or being influenced by others and being beyond complete control (Misztal 2012, 217–218; Quepons 2020, 5). Due to (2) insufficient anticipation and prediction of future events, we are unable to weigh up all potential possibilities and show ourselves to be vulnerable to the unforeseen. Last but not least, (3) past experiences or traumas determine which relationships we enter into now or in the future and reveal a vulnerability that has already been shaped (Misztal 2012, 218–219; Quepons 2020, 5).

The previous considerations illustrate (1) the subjective character of being vulnerable, insofar as people must ascribe this status to themselves or feel vulnerable (Quepons 2020, 4–5). The (2) objective aspect is that practically all people are vulnerable (Delor and Hubert 2000, 1560–1561), which requires further investigation both for the general use of this term and its applicability in the context of trust. In contrast to the general determination of vulnerability on the basis of being human, the person‐ or group‐specific attribution (Adger 2006, 276–277; Kottow 2004, 283–284) could take on ethically questionable forms if people are labelled as vulnerable without leaving this assessment to the respective people themselves (Beauchamp and Childress 2019, 286–288). In addition, (3) a logical connection between vulnerability and trust becomes apparent, insofar as one's own vulnerability can lead to trusting or not trusting other people (Pellegrino 1994, 483–484). According to the previous explanations, a distinction can be made between a social attribution and a human constant with regard to vulnerability, which Kottow describes as *susceptibility* and *vulnerability* (Kottow 2004, 285–286).

Although vulnerability is explicitly or implicitly regarded by many authors as an essential starting point for trust, an even stronger focus on this concept has emerged in recent years (Baghramian et al. 2020, 576–577; Mah et al. 2023, 2–3). Misztal expresses the need to be able to rely on other individuals on the basis of our vulnerability: ‘[…] human vulnerability […] makes trust fundamental to our lives […]’ (Misztal 2012, 209). This refers to the second form of vulnerability, which basically affects all people. However, with regard to the following analysis, the question arises as to whether this human constitution can actually be equated with that vulnerability in trust relationships and what consequences are associated with it. In the subsequent investigation, the question is explored as to what extent one's own vulnerability and the vulnerability perceived by oneself (Adger 2006, 276) are related to the trust between humans and care robots. It will be shown that this vulnerability is not only to be seen as something threatening, seemingly imposing trust and thus promoting the heteronomy of humans, but that there are also positive aspects associated with it.

With a view to the following examination, the essential link between trust and vulnerability should already be emphasized at this point. Nienaber et al. also make this clear in their study when, according to their explanations, a distinction must be made between (1) the *willingness* to show vulnerability and (2) the *vulnerability* given at a specific point in time, which, according to the order given, go hand in hand with (1) the intention to trust and (2) the trusting behaviour (Nienaber et al. 2015, 572). This classification shows that this idea of vulnerability does not merely describe a dependence on other people, but rather that it also brings to light something like a subject‐related quality. This seems to run counter to the common—media and socially constructed (Misztal 2012, 211–212)—view of *being vulnerable*, because it is not about a kind of neediness, whereby one is dependent on the benevolent and caring behaviour of others. The decisive factor is the personal willingness to trust others—on the basis of good reasons—which can certainly be used to counter aspects of one's own vulnerability.

## 3 Trust and Mutual Dependence

After considering vulnerability, attention shifts to trust and the extent to which *being vulnerable* can also be understood in a positive way. To this end, the types proposed by Misztal are related to trust and discussed on the basis of care robotics. The concept of trust is usually associated with a form of vulnerability (Baghramian et al. 2020, 575–576; Whyte and Crease 2010, 411–413), because the trusting subject is dependent on the actions of the respective counterpart—and therefore also vulnerable. As a result of this dependence, a certain degree of control is transferred to the person who is trusted (Misztal 2019, 46–48). In this context, the interpersonal interdependence that Misztal previously described in terms of human social dependence comes to light. Nevertheless, such a trust constellation is not an externally imposed constraint, as it is rather assumed that the addressee of the trust also feels obliged to comply (Faulkner 2017, 109–111; Thompson 2017, 644–646). This is associated with a personal conviction, which is based on rational, normative and emotional aspects, and in a certain way refers to the object of trust.

From a rational point of view, for example, anticipated reasons such as sincerity, reliability, competence or previous constellations speak in favour of trust in a particular addressee (Plomp and Ballast 2010, 263–265). From a normative point of view, a certain form of ought can be derived from these considerations to follow precisely these rationally understandable and insightful aspects. The emotional dimension can be explained in particular by the relationship level, because trust in a respective counterpart is hardly only associated with good reasons, but rather with affective criteria on an emotional level, which have a motivating force. In a comparable way, this was described by Quepons (2020, 1) as an essential dimension of one's own vulnerability, or also by Nienaber et al. (2015, 575–576) with active vulnerability. One could take the position that, based on the previous considerations, the vulnerable situation is consciously accepted for the sake of trust.

Trust can be understood, for example, to include the trustworthy subject as the basis for the *dependency* assumed, or to refer to the aspects associated with the trust constellation (Budnik 2016, 103–105). Most academic literature differentiates between *trust* and *mere reliance*. According to Ryan, the first view would not be applicable to care robots because objects can only be relied upon—they lack the status of moral actors (Ryan 2020, 2758–2760). Thompson's considerations go in a similar direction, according to which the distinction between feeling *betrayed* and merely *disappointed* refers to the two previous differentiations, but care robots can (currently) only be located in the second category (Thompson 2017, 650–652). In this context, reliance is associated with a pure probability of behaviour, whereas the concept of trust implies more (Budnik 2016, 105–107; Hartmann 2010, 20–22). Within the relational structure, this more refers to the connection between the normative component as a moral ought, as well as an affective and rational dimension (Kropf 2025, 2; Ryan 2020, 2753–2755; Thompson 2017, 652–654).

With regard to the essential care robots in this article, the question may arise as to whether the affective or normative components can be fulfilled by AI‐based robots at all (Ferrario et al. 2020, 527–529). Apart from this, it is plausible to agree with Nguyen that the distinction between being *disappointed* and feeling *betrayed* can be viewed critically (Nguyen 2022, 217–220). Decisive for the following considerations are trust constellations that refer to human–robot interaction. Personal experience has a significant influence on trust, which makes the rather sceptical attitude towards technical helpers understandable (Gilpin 2022, 143–144; Green 2018, 212–214; Lenk 2010, 32–34). Luhmann speaks of a familiarity that is required as a necessary basis for trusting relationships, so to speak, which also involves time, a fundamental understanding and moral considerations (Luhmann 1989, 18–20). Nguyen even refers to an unquestioning attitude that has set in if one should trust—possibly also certain objects (Nguyen 2022, 219–222).

Possible relations of trust are either (1) *A trusts B for a valuable good X*, or (2) only *A trusts B*. Care robots^1^ are increasingly being integrated into the care sector in order to relieve the burden on professionals in terms of time and resources, to implement innovative forms of interaction or to make care itself better (Kabacińska et al. 2021, 919–920; Zafrani et al. 2023, 1–2). Although this better has yet to be proven in reality, some studies (Poulsen and Burmeister 2019, 7–9; Poulsen et al. 2018, 323–325; Schönmann et al. 2023, 1017–1018; Yew 2021, 641–642) not only speak in favour of the general acceptance of technical companions, but also illustrate the promotion of well‐being, social and physical engagement, or even the reduction of stress and depression. For example, a person in need of care could trust that the AI‐based robot is telling the truth. This corresponds to the first variant (three‐digit) and seems quite conceivable and justified under certain conditions. However, mere trust in the sense of the second form (two‐digit) is unimaginable for many authors, among other things because of the lack of judgement, a capacity for reflection or an ethical ability to analyze (Budnik 2016, 113–115; Hawley 2014, 2–3; Ryan 2020, 2763–2765). Regardless of whether, and if so in what form, trust in these technical entities is possible, the reference to the dependency mentioned by Misztal (Misztal 2012, 220–221) appears to make sense.

If we, as human beings, find ourselves in a social structure, our own actions and decisions are to a certain extent dependent on other individuals—and we also co‐determine their creative spaces—which reveals a reciprocal relationship (Petherbridge 2021, 2–3). Similarly, the actions of an AI‐based robot also have certain effects on affected individuals. A machine that wants to encourage a group of people in need of care to exercise more will play certain algorithmically anchored protocols, simulate physical movements or communicate verbally with others. On the one hand, the trust required to join this group as an affected person would promote vulnerability, because the technical companion or its actions could abuse or violate the trust placed in it. On the other hand, the already existing human vulnerability seems to be a decisive factor in trusting, as this creates new opportunities. Misztal describes it similarly, because ‘[…] trust needs to be seen as offering both, a solution to the problem of our vulnerability, and as exposing us to more risks’ (Misztal 2012, 216).

However, it is important to answer what exactly is meant by these *additional* possibilities. When we trust other people, or in this case technical companions, we do so in the knowledge that the action expressed through trust does not necessarily have to be performed by ourselves—or at least that we do not want to. If a person in need of care trusts a robot to help them out of bed, bring them their medication or read them a story, this not only reflects a vulnerability that promotes or causes trust. At the same time, something new can be gained through a form of trust. In the example given, this gain refers to what is read aloud, physical help or medicalization. This is a consequentialist approach, whereas the focus on trust itself places the person—or the robot—at the centre. The trusting attitude can make the object of trust feel obligated to adhere to the associated goals and values, thus promoting a lasting trust relationship (Baier 2001, 45–46). In this sense, trust seems to enable the promotion of social relationships, the provision of additional options and the reduction of one's own vulnerability.

While the consequences of the additional options and vulnerability reduction can also be applied to AI‐based care robots, the promotion of a social relationship seems difficult to understand at first glance. Particularly because the idea of a relationship that we are familiar with is often accompanied by reciprocal (human) obligations, or the ‘[…] bonds of solidarity […]’ (Misztal 2012, 223) described by Misztal. Modern care robots are not currently capable of having human feelings, ethical reflection or empathy. Apart from that, humans sometimes enter into social relationships with other people without taking the interests of the other person seriously. This attitude can be prevented in technical assistants, at least in theory, by adequate programming (Campbell 2020, 112–113; Cifuentes et al. 2020, 61–62; Kabacińska et al. 2021, 925–926). In addition, the integration of technical aids into inpatient facilities and their interaction with people requiring care offers additional options that could not be implemented by human care professionals. On the one hand, this addresses the inherent aspects of the machine (physical performance, programming, appearance) (Langer et al. 2019, 233–234) and, on the other hand, the specific concept (less intentional abuse, less moral personality) of AI‐based trust, which cannot be equated with the conventional concept of trust.

From the previous considerations, it becomes clear that trust in technical companions can also reduce one's own vulnerability, in the sense of a basic human constant, and thus shape the reciprocal relationships mentioned by Misztal (2012, 222–223) even more strongly—which also has positive effects. What this is intended to show is that interdependencies do not necessarily have to be seen as something that reveals human vulnerability and also produces certain susceptibilities. After what has been said so far, it is equally plausible to claim that trust in the abilities, the sensory perceived normative claim and the algorithmically anchored importance of the addressee of trust can contribute to a reduction in one's own vulnerability. Sweeney also advocates the willingness to trust, also because of the social relevance of technical companions (Sweeney 2023, 423–424). For example, a care robot helps an elderly person out of bed, which provides additional options for action—getting out of bed—and thus also reduces the person's own vulnerability. The more this human–robot interaction establishes itself as a specific relationship, the easier it will be to establish trust, which may steadily reduce the vulnerability that corresponds to human nature.

## 4 Trust and Unpredictability

After considering vulnerability on the basis of the human–robot *relationship*, the focus is on the anticipatory problem described by Misztal (2012, 224–225). Social coexistence would be characterized by constant fear, hostility, uncertainty or even a possibly unfounded caution if people did not trust each other with at least a minimal understanding (Faulkner 2017, 111–114; Misztal 2019, 45–47). As already described, trust is a relational construct that involves not only descriptive terms, but also normative expectations (Petherbridge 2021, 4–5; Yew 2021, 633–635). When people trust a counterpart, they usually do not do so on a whim, but rather because there are good reasons, or at least a reason, for them to hand over their own options for action to another individual to a certain extent (Baier 2001, 42–44; Kropf 2025, 3). This is also linked to the indeterminacy of the future mentioned by Misztal or the impossibility of anticipating all conceivable scenarios and outcomes as such (Misztal 2012, 223–224). Nevertheless, the potential assumption of this vulnerability seems to make it possible to hand over responsibility, which to a certain extent transfers vulnerability.

This transfer of responsibility means that the person placing trust not only places options for action in the hands of another person—or in this case the technical processes of the machine—but also expects something as a result. This expectation can be reconciled with the previous considerations and refers to the conviction that the respective addressee of the trust also feels obliged to comply with the associated goods to be secured (Hawley 2014, 8–9; Luhmann 1989, 24–25). This duty corresponds to a moral responsibility, as the entrusted goods are certain things with moral content, such as human life, health or freedom. These important goods form a decisive basis not only for the relationship of trust, but also for the assumption of moral responsibility. By recognizing (rationally) and being motivated (emotionally) to carry out a certain action or to show responsibility, we express our respect for the respective counterpart and the central goods.

Moral responsibility often implies a form of justification to explain one's actions and decisions to other people, groups or even to oneself. An action or decision that leads to harm and is insufficiently justified may result in blame, whereas actions or decisions that are morally praiseworthy may promote praise. Moral responsibility therefore implies not only descriptive terms, but also normative expectations to meet the associated requirements (Menges 2020, 11–12; Shoemaker 2017, 485–486). Even if this responsibility is usually only attributed to moral actors, this consideration can be extended to the integration of care robots, at least to a certain extent. In particular, the role responsibility mentioned by Vincent (Vincent 2011, 17–18) seems plausible here, insofar as the technical companions have an explicit role in a care home. A widely accepted formulation for moral responsibility is: A is responsible for X, towards B, with reference to Y (Werner 2002, 521–522). Although this representation appears in descriptive form, normative expectations are crucial, which may allow the transfer of human vulnerability.

If a person in need of care in a specific facility increasingly comes into conflict with a particular care professional, the involvement of a technical companion can be useful. Trust in the moderating and mediating skills of the care robot—which are confirmed by empirical data (Chita‐Tegmark and Scheutz 2021, 205–207; Zafrani et al. 2023, 2–3)—can help to delegate personal responsibility in these delicate scenarios. The care robot (A) is then technically responsible for mediating (X) and moderating communication between the caregiver and the person concerned. For example, the values (Y) anchored in the programming, such as respect, life, health or equal cooperation, would be decisive for this, whereby the machine takes these aspects into account within the interaction. Conflicts can be resolved or even prevented by asking specific questions, using adequate language for the situation and adopting a neutral position (Chita‐Tegmark and Scheutz 2021, 205–206; Petherbridge 2021, 6–7). The corresponding counterpart (B) is then theoretically one of the two persons addressed, but in reality, will refer to the algorithmic specifications. On the one hand, the technical companions are able to act on the basis of a given protocol and show behaviour, but on the other hand, a corresponding justification does not currently seem feasible (Yew 2021, 632–633)—which means that the responsibility *towards B* reaches technical limits.

What can be recognized is a transfer of responsibility to the person or, in this context, the machine^2^ that is trusted. *Trust* can therefore not only be justified with reference to the vulnerability of the human being, but rather a form of human vulnerability can be transferred through the delegation of responsibility. The person in need of care in the previous example no longer has to take care of resolving the conflict themselves, but hands this task over to the technical helper, so to speak. The previously existing vulnerability, also in the sense of a personally perceived responsibility for resolving the conflict, is thus shifted to the object of trust. With regard to the unpredictability of future behaviour of other beings mentioned by Misztal (2012, 224–225) and the associated importance of mutually valid promises, it should be noted here that an assessment of the machine's actions can be much more meaningful and reliable than, for example, with a human counterpart (Oksanen et al. 2020, 6–8). These machines operate within a predefined algorithmic‐structured protocol and cannot be misled by human inclinations, sensitivities or even malice.

In addition, the aforementioned necessity of a mutually accepted promise loses significance because non‐compliance usually requires intentional action, which does not currently correspond to the scientific state of robotics (Giorgi et al. 2023, 8–9; Langer et al. 2019, 235–236; Naneva et al. 2020, 1194–1196). These machines can also make mistakes, fail to meet certain expectations or cause harm, but not in a deliberate kind of way. The previous considerations make it clear that trust in technical companions is also tantamount to a delegation of responsibility and thus the existing vulnerability, for example, in the sense of a task to be completed with anticipatory difficulties, is transferred. For example, those who hand over responsibility for a certain task cannot be prosecuted or morally blamed for non‐compliance (Werner 2002, 522–523). Thus, trust is once again associated with positive consequences, which are represented in a transfer of personal vulnerability as well as responsibility and, with regard to the care robots, take into account the additional aspect of (impossible) anticipation in a specific way.

## 5 Trust and Experiences

After the first two types—relationships (1) and the anticipation problem (2)—have been examined in Misztal's presentation of vulnerability, the main focus is on the third type. Here, past experiences (3) or traumas play a decisive role in whether and in what way a current or future trust constellation is entered into at all (Misztal 2012, 225–226). In this context, it is conceivable that people do not easily decide to trust other people if they have previously experienced disappointments in this regard. At the same time, it will be much easier for people to trust others if positive experiences are frequently associated with this trust. The technical companions now show that although potential trust often appears to be associated with a reduction in freedom, this can also be perceived differently. In particular, because the concept and the associated decision to trust others is something *voluntary*, which, as already described, does not represent external coercion.

In care facilities, for example, a care robot is trusted to fetch bandages or prepare medication. In order to avoid mere dependence on the machine, moral points of reference are required, which are represented by the relationship and thus also express the entrusted good. Baier also describes this as a benevolent form of *caring* (Baier 2001, 46–48). The affective component is thus recognizable and makes it clear that trust is not accompanied by exclusively rational considerations. Rather, there is a conviction on the part of both the trusting person and the robot—in an algorithmically anchored and sensorially recorded form—that something morally important is associated with it (Thompson 2017, 650–652). For Hartmann, trust is like a treat, a reward or a privilege that not everyone can enjoy (Hartmann 2020, 97). This idea emphasizes the significance of the experiences mentioned by Misztal (Misztal 2012, 233), which, although not the sole determinant of trust, play a notable and *predictive* role.

Due to the increasing—and not universally established—use of robots in care homes, it should also be noted that specific experiences and encounters with these technical aids are limited. In particular, personal contact (Honekamp et al. 2019, 61–63; Plomp and Ballast 2010, 267–268) and familiarity with the potential recipients of trust can promote corresponding behaviour. For many people, however, it is a reality that interaction with a care robot represents something new, unfamiliar and possibly also threatening. For this reason, it makes sense in this context to consider technical aids in other areas that are of relevance to individuals. Examples include electric cars, robotic support systems in the home (vacuum robots) or everyday life (lawn robots), technical assistants such as Siri or Alexa, or the meaning of a general affinity for technology for individuals. People with an affinity for technical devices will also react with fewer reservations towards care robots than comparable people who are less interested in technology (Papadopoulos et al. 2023, 7–8).

Apart from that, previous life events, the memories and experiences associated with them, shape the kind of trust we place in others (Quepons 2020, 5–6). This already established and shaped vulnerability cannot simply be turned off or erased, because it makes us the people we are, so to speak. In most cases, mistrust will be particularly difficult to overcome (Plomp and Ballast 2010, 267–268). However, the reference to care robotics and the associated actors offers a new and innovative approach, because these technical helpers will trigger certain memories in very few people—because they are still missing. Several studies (Chita‐Tegmark and Scheutz 2021, 210–211; Schönmann et al. 2023, 1016–1017; Soljacic et al. 2024, 698; Sweeney 2023, 424–425) show the extent to which people in need of care also feel comfortable with robots and prefer them to their human counterparts in certain activities. With their specific technical appearance and different nature—compared to humans—trust constellations with care robots are possible in a *different* way. Although past vulnerability also plays a role in human–robot interaction that should not be underestimated, its limitations also offer the potential for a new approach.

The willingness to trust an AI‐based robot requires a voluntary decision from the people concerned. This demand for voluntariness refers to a phenomenon that has already been addressed but not discussed enough. Trust in certain addressees is not only accompanied by a potential vulnerability based on *their* decisions, but also by the restriction of freedom—at least according to the view often found in academic circles (Faulkner 2017, 110–112; Hawley 2014, 10–11; Misztal 2019, 46–48). Anyone who relies on the actions of a care robot through a given trust, for example, gives up a certain scope of action in that the goods associated with the trust relationship and their realization are dependent on the algorithmically simulated decisions of the technical assistants (Sweeney 2023, 424–425). In addition to this plausible and logical conclusion, however, it is important to bear in mind that trust not only represents a reduction of freedom, but can also be understood as an *explication* of freedom. This reveals the possibility of a trusting person to follow their own considerations, context‐ and person‐specific assessments or even previous experience.

Only through these subject‐related processes—which integrate cognitive, affective and normative (Hawley 2014, 11–12; Thompson 2017, 650–651) aspects—can it really be said that trust expresses the freedom of the subject. Consequently, certain goods, people considered important or even one's own life, are placed in the hands of another person. In this context, this personal conviction of being able to trust a technical companion in specific situations, for example, is also crucial, which can be linked to the *intentionality* described by Beauchamp and Childress (2019, 102). In many cases, we will trust other people, or in this context a machine, without making lengthy and time‐consuming considerations as to whether it is justified from a rational point of view. In most cases, the previously mentioned experience (Misztal 2012, 219–220), previous encounters or past assessments have established a relationship of trust that facilitates future decisions in this regard.

However, this aspect of the intentional decision by no means slips into insignificance, as the trusting subject will decide in one way or another in the sense of a planned and considered approach (Beauchamp and Childress 2019, 102–103). Even if such processes of deliberation will take place quite quickly in reality—also due to the experience mentioned above—it is much more implausible to claim that they do not take place. Anyone who trusts another person or even a care robot without having certain convictions regarding the positive outcome of the trust relationship, which are also reflected in the form of intentional action, can hardly use the term *trust*. On the one hand, it would be irrational to trust at all due to the lack of conviction and, on the other hand, it is part of the concept of trust itself that such considerations take place or have taken place. Trust in a specific addressee is therefore something special, as Hartmann also emphasized (Hartmann 2020, 97). Not every interaction or relationship allows us to speak of trust at all, because certain convictions must be present on the part of the trusting person. However, these in turn cannot be justified without the object of trust or the relationship itself, which once again illustrates the intentional dimension (Plomp and Ballast 2010, 267–268).

It can be seen from the previous descriptions that intentional trust in an object of trust is associated with a specific form of freedom, which can also be seen as a gain or at least something positive. If a bedridden person trusts their care robot to gently lift them out of bed, then this attitude is both intentional and an explication of freedom. This means that personal considerations and convictions can only be realized and expressed through the trust placed in a respective counterpart: ‘[…] trust gains its fundamental meaning as an active becoming vulnerable toward others’ (Quepons 2020, 9). In this formulation, the *active* seems to refer to the importance of personal freedom. Intentional and convinced trust thus not only leads to potential vulnerability, but also expresses the freedom of the trusting subject. This explication of freedom also appears to be of ethical relevance, insofar as it brings to light the trustworthiness of the other person or the importance of the relationship. In addition, it is possible for the trusting subject to follow their own convictions and thus make personal ideas and values realizable.

With regard to the traumas mentioned by Misztal (2012, 218–219) or earlier experiences, this positive approach to the concept of trust not only allows people to *freely* pursue their own convictions about who exactly should be trusted and why. Rather, it can also be concluded from the previous considerations that this third type of vulnerability can be overcome through an autonomous and freedom‐explicating trust. Although traumatic experiences or difficult life events prospectively shape our willingness to trust others, a trust full of conviction, so to speak, can lead to overcoming this already existing vulnerability and promote personal growth. One could also say that trust ‘[…] results not only in a recognition of our vulnerability but in a becoming vulnerable in an active and positive sense’ (Quepons 2020, 8). While this positive form is certainly in line with the previous descriptions, it is not only about the shift in vulnerability, but rather about the possibility of leaving behind this already internalized vulnerability through trust—also in an AI‐based robot.

## 6 Final Considerations

In this article, the connection between vulnerability and trust was examined from an ethical perspective. The vulnerability of the human being could be described in terms of group affiliation on the one hand and as a basic human condition on the other. Above all, the three types of vulnerability proposed by Misztal (relationships, anticipation problems, traumas) were the starting point for the investigation of the concept of trust and the associated thoughts. The consideration of AI‐based care robots seems particularly reasonable and ethically relevant due to demographic change and the technologization of care in general. Even if many authors consider trust in these technical companions to be simply unimaginable, their use could illustrate the potential possibilities of accepting one's own vulnerability or potential trust in these actors. What is necessary here is an approach that considers a technical form of trust to be quite plausible and does not allow itself to be influenced by anthropocentric reservations.

The key findings of this previous investigation include (1) the additional options, (2) the potential transfer of responsibility and (3) the explication of freedom through trust—possibly also in technical companions. The (1) additional options open up certain courses of action for the trusting person that would not be possible without the involvement of care robots, for example, which can also reduce their own vulnerability. The (2) delegation of one's own duties or responsibilities consequently also leads to the associated vulnerability being transferred to the object of trust, which can be explained in this context by the technical companions. Last but not least, (3) the intentional and conscious attitude of trust can lead to overcoming one's own vulnerability, possibly characterized by bad life events, and thus also express the freedom of the respective individual.

## Author Contributions

The author has accepted responsibility for the entire content of this manuscript and approved its submission.

## Ethics Statement

The author has nothing to report.

## Consent

The author has nothing to report.

## Conflicts of Interest

The author declares no conflicts of interest.

## Data Availability

The author has nothing to report.

## Endnotes

1 This article focuses in particular on socially assistive robots (SARs), which can not only perform physical assistance tasks such as lifting or transportation. Rather, these technical companions are assigned to socially and physically motivate people in need of care and integrate them into an existing environment. Their tasks and possible applications are diverse, their technology is based on machine learning in many cases and there is great potential for further development (Kropf 2025, 9; Langer et al. 2019, 231–232).

2 Ultimately, care professionals will have to assume responsibility because care robots are not moral actors. The decisive factor in this context is rather the transfer of this moral responsibility perceived by the person concerned, for which the technical companion assumes a representative function or even the middle position before the care professional (Yew 2021, 640–641).
